# Target Cell APOBEC3C Can Induce Limited G-to-A Mutation in HIV-1

**DOI:** 10.1371/journal.ppat.0030153

**Published:** 2007-10-26

**Authors:** Khaoula Bourara, Teri J Liegler, Robert M Grant

**Affiliations:** 1 Gladstone Institute of Virology and Immunology, University of California San Francisco, San Francisco, California, United States of America; 2 Department of Medicine, University of California San Francisco, San Francisco, California, United States of America; University of Pennsylvania School of Medicine, United States of America

## Abstract

The evolutionary success of primate lentiviruses reflects their high capacity to mutate and adapt to new host species, immune responses within individual hosts, and, in recent years, antiviral drugs. APOBEC3G (A3G) and APOBEC3F (A3F) are host cell DNA-editing enzymes that induce extensive HIV-1 mutation that severely attenuates viral replication. The HIV-1 virion infectivity factor (Vif), expressed in vivo, counteracts the antiviral activity of A3G and A3F by inducing their degradation. Other APOBECs may contribute more to viral diversity by inducing less extensive mutations allowing viral replication to persist. Here we show that in APOBEC3C (A3C)-expressing cells infected with the patient-derived HIV-1 molecular clones 210WW, 210WM, 210MW, and 210MM, and the lab-adapted molecular clone LAI, viral G-to-A mutations were detected in the presence of Vif expression. Mutations occurred primarily in the GA context and were relatively infrequent, thereby allowing for spreading infection. The mutations were absent in cells lacking A3C but were induced after transient expression of A3C in the infected target cell. Inhibiting endogenous A3C by RNA interference in Magi cells prevented the viral mutations. Thus, A3C is necessary and sufficient for G-to-A mutations in some HIV-1 strains. A3C-induced mutations occur at levels that allow replication to persist and may therefore contribute to viral diversity. Developing drugs that inhibit A3C may be a novel strategy for delaying viral escape from immune or antiretroviral inhibition.

## Author Summary

HIV has shown a chameleon-like nature, always changing to adapt to its environment. Defining the factors that drive and regulate genetic changes in HIV over time is key to understanding how HIV causes disease and escapes from the body's immune responses and drug treatment. The diversity of HIV has implications for the development of effective drugs and for fostering better immune responses. In this study, we showed that a human protein, called APOBEC3C (A3C), could induce certain mutations in some HIV-1 strains, including those derived from a patient who had developed rapid drug resistance. Eliminating the expression of this protein prevented the mutations in two different types of cells. Furthermore, short-term A3C expression was sufficient to cause mutation. We conclude that A3C is necessary and sufficient to induce signature mutations in HIV-1. A3C-induced mutations may provide potential benefit for the virus if the mutation rate is low enough such that the majority of viruses are able to replicate, while accumulating a limited number of novel mutations that may allow the virus to survive in the face of antiviral drugs or immune responses.

## Introduction

The evolutionary success of primate lentiviruses is evident from their prevalence in Old-World primates and their capacity to spread to new host species, frequently leading to the emergence of zoonotic disease [1,2]. Establishing persistent infection in individual hosts requires high mutation rates and rapid and extensive viral adaptation, which allows the virus to escape from humoral and cell-mediated immune responses [3,4]. Rapid viral adaptation also produces drug resistance that limits the effectiveness of therapy in many patients. Thus, understanding lentiviral genetic variation is crucial for HIV therapy.

An important mechanism of HIV genetic variation is G-to-A mutation during reverse transcription [5,6]. Such mutations can be mediated by a family of DNA-editing enzymes with a strong preference for specific dinucleotide contexts [7–10]. For instance, APOBEC3G (A3G) induces high frequency of GG-to-AG mutations [11–15] whereas APOBEC3B (A3B) and APOBEC3F (A3F) cause GA-to-AA mutations [7,11,12]. In contrast, APOBEC3C (A3C) acts on both GA and GG dinucleotides, with a preference for GA over GG [7,12,16]. G-to-A mutations have been detected in at least 43% of HIV-1-infected patients, indicating that such mutations occur in a setting of persistent replication [17].

A3G- and A3F-induced mutations are suppressed by HIV-1 virion infectivity factor (Vif), which is typically expressed in vivo, thus limiting their contribution to the adaptation of viral populations [15,18–22]. In contrast, A3B and A3C are relatively resistant to the effects of Vif [7,16,23], which suggests they may play a role in HIV diversity. However, unlike A3C [24], A3B is not expressed in the lymphoid cells that serve as targets for HIV-1 infection, which limits its potential role in the evolution of wild-type HIV-1 [9,23].

We hypothesized that G-to-A mutation in *vif*-expressing viruses is caused by an APOBEC that does not have a strong antiviral activity, is relatively resistant to Vif action, and is expressed in HIV-1 target cells. To contribute to the adaptation of viral populations, the APOBEC activity should be weak/moderate, such that mutations are sufficiently infrequent to allow some mutant progeny to survive. APOBEC3C (A3C) is a candidate because it is expressed in cells targeted by HIV-1, including PBMC, macrophages, and thymocytes [24]. A3C can cause mutations in the GA and GG contexts [7,12]. A3C has diverged by at least 40% from the A3G, A3F, and A3B, which are known to have antiviral activity against HIV-1 [7,9,24,25]. Furthermore, a recent report showed that APOBEC3C exerts potent antiviral activity against simian immuno-deficiency virus, but not to a lab-adapted clone of HIV-1 [16]. Low-level activity of DNA-editing enzymes would allow some HIV-1 progeny to be viable enough to be selected by immune responses or antiviral drugs.

In this study, we assessed the influence of A3C on G-to-A mutation and HIV-1 replication using multiple infectious viruses differing in Gag and/or Pro. The first group of isogenic *vif-*expressing NL4–3 molecular clones (210WW, 210WM, 210MM, 210MW) contain combinations of pre- (W) and post-therapy (M) *gag* and *pro* genes from an HIV-1-infected patient who rapidly developed resistance to a protease inhibitor–containing regimen [26]. A second group of drug-resistant viruses includes three NL4–3-based molecular clones with point mutations introduced in the protease and reverse transcriptase (RT) genes. NL4–3 and LAI were also included as controls.

## Results

### A3C Expression Is Required for G-to-A Mutation in Magi and 293T Cells

**Figure 1:**
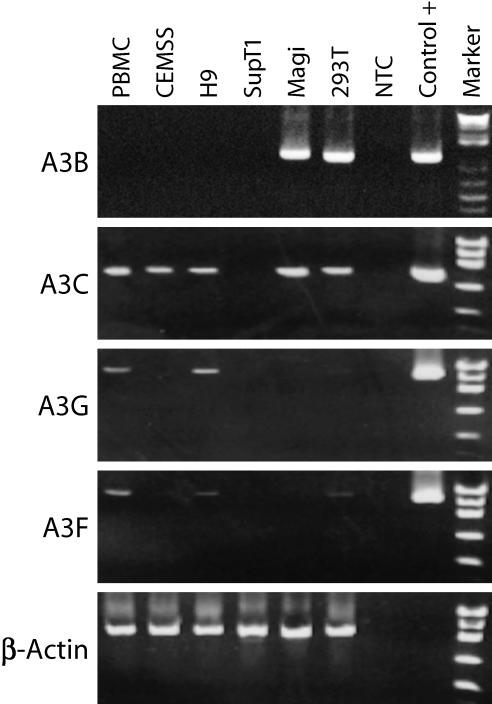
Expression of APOBEC mRNAs in Different Cells Poly A+ RNA was isolated from individual cell types and APOBEC reading frames (A3B, A3C, A3F, and A3G) were amplified with primers specific for a single ORF. The specificity of the primers was verified by sequencing the PCR products. RT-PCR reactions for A3B, A3C, A3G, and A3F ORFs were analyzed by agarose gel electrophoresis. PCR templates were cDNAs for the indicated cell types or plasmids specific for A3B, A3C, A3G, or A3F (positive controls). Water was the nontemplate control (NTC), β-actin was the internal control, and reactions lacking reverse transcriptase (RT) were also used as control during cDNA preparation (unpublished data).

**Figure 2:**
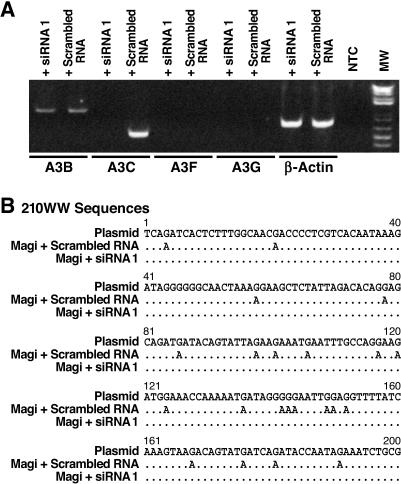
Inhibition of A3C Expression and G-to-A Mutation by siRNA 1 (A) Magi cells were transfected with siRNA 1 (50 nM) against A3C or with scrambled RNA. A FITC-conjugated oligo was cotransfected and FITC-positive cells were sorted 48 h following transfection. PolyA+ RNA was isolated, and RT-PCR was performed with primers specific for A3B (lanes 1 and 2), A3C (lanes 3 and 4), A3F (lanes 5 and 6), and A3G (lanes 7 and 8). β-Actin (lanes 9 and 10) was used as an internal control. A sample lacking template DNA (lane 11) was used as a negative control. Lane 12 is a molecular weight standard. (B) The transfected cells were infected with the VSV-G-pseudotyped 210WW and collected 24 h later. Viral DNA was amplified using the sensitive mutation assay and population sequencing was performed to analyze G-to-A mutation. The G-to-A mutation typically appeared as a mixture of G and A peaks at a given position, with G, the wild-type sequence, as the predominant peak. A change from G-to-A was considered a true mutation only if A represented at least 20% of the peak.

To determine whether A3C is responsible for inducing G-to-A mutation in HIV-1, we inhibited A3C expression in HIV-1-infected Magi cells and examined the viral sequences. Magi cells, which normally express A3C (Figure 1), were transfected with siRNA 1 targeting A3C RNA at position 167–185 relative to the start codon. A FITC-conjugated RNA oligo was cotransfected to mark the transfected cells. FITC-positive cells were sorted 48 h after transfection. We found that no A3C mRNA was detectable in FITC-positive cells transfected with A3C-specific siRNA (Figure 2A, lane 3) after 48 h in culture. By contrast, cells transfected with a control, scrambled RNA, had normal A3C mRNA levels. The cells were then infected in a single-round replication assay with NL4–3 and 210WW viruses produced by 293T transfection (both virus stocks were p24-normalized, delta *env* and VSV-G-pseudotyped). After an additional 24 h in culture, cellular DNA and RNA were extracted. A3C mRNA was undetectable by RT-PCR (unpublished data).

The presence of G-to-A mutations in NL4–3 and 210WW was then examined using a sensitive mutation assay adapted from Janini et al., 2000 [17] and designed for detecting G-to-A viral mutants over a more abundant wild-type background. This assay sensitively detects G-to-A mutants in the viral population and measures the relative frequency of mutation in the GA versus GG contexts (see Materials and Methods for a detailed description).

We found no G-to-A mutation in virus grown in Magi cells transfected with the A3C siRNA 1 (Figure 2B). By contrast, G-to-A mutations were detected in a parallel experiment where Magi cells were transfected with scrambled RNA (Figure 2B), indicating that A3C can induce G-to-A mutations in 210WW. The dinucleotide context of G-to-A mutation also correlates with the activity of A3C since both GA and GG contexts were modified with GA relatively more preferred than GG (17 GA over five GG, Figure 2B). Surprisingly, no G-to-A mutations were detected in NL4–3 infections of Magi cells transfected with the siRNA 1 or with the scrambled RNA (unpublished data).

To confirm that the siRNA 1 was specific to A3C, we examined the expression profile of other APOBEC genes. We found that the Magi cell line did not express mRNA for A3G or A3F, either before or after siRNA treatment (Figure 2A, lanes 5–8). Magi cells do express A3B [27], but A3B mRNA levels were not altered by the A3C siRNA treatment (Figure 2A, lanes 1 and 2) as expected because there were nine nucleotide mismatches between the A3C siRNA 1 and A3B mRNA sequences.

To ensure that the suppression of G-to-A mutations in siRNA 1–transfected cells is not due to an off-target effect of the siRNA, we repeated these experiments with a second siRNA (siRNA 2), which targets residues 107–125, and causes a partial knockdown of A3C mRNA expression (Figure S3). When transfected into 293T cells, endogenous A3C mRNA levels were reduced by 74% and the frequency of G-to-A mutations was correspondingly reduced by 70% compared to the control scrambled RNA. siRNA 2 was specific for A3C mRNA; no reduction in A3B, A3F, and A3G expression was observed compared to the scrambled control.

Thus, specific inhibition of A3C expression by siRNA eliminated 210WW G-to-A mutations after infection of Magi (Figure 2B) and 293T cells. This result suggests that A3C expression is required for the induction of G-to-A mutations in 210WW.

### A3C Expression in SupT1 Cells Is Sufficient for G-to-A Mutation

**Figure 3:**
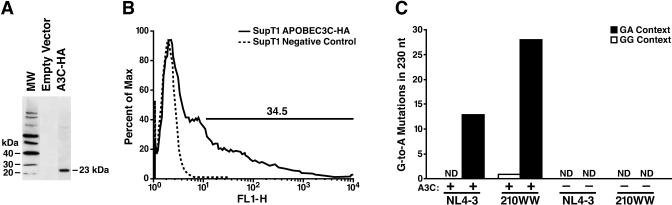
Effect of A3C Expression in SupT1 (A) The A3C gene cloned into the pTT-IRES-GFP lentiviral vector with an HA tag was expressed in 293T cells, and the protein detected with a polyclonal HA antibody. (B) This construct was used to transfect SupT1 cells. After 48 h, transfection efficiency of the construct was monitored by FACS analysis of GFP expression. (C) Transfected SupT1 cells were infected with the VSV-G-pseudotyped 210WW and NL4–3 HIV-1 and collected 24 h later. Viral DNA was amplified using the sensitive mutation assay, and population sequencing performed to analyze G-to-A mutation. The total number of G-to-A mutations in the protease region (positions 2255–2485) was counted separately for the GA and GG contexts. No mutations were detected after infection of SupT1 cells transfected with an empty vector. ND, non-detectable mutations.

To further confirm that A3C in the target cells was responsible for the G-to-A mutations, the A3C gene was transfected into SupT1 cells, which do not express A3C mRNA (Figure 1). A3C cDNA was cloned into an expression plasmid that expresses the bicistronic A3C-IRES-GFP under the R PGK promoter and carries the influenza hemaggluttinin (HA) epitope tag (see Materials and Methods). A3C expression in 293T-transfected cells was confirmed by western blotting with a polyclonal antibody specific for HA, displaying the predicted electrophoretic mobility of 23 kDa (Figure 3A). The A3C expression plasmid was then transfected into human SupT1 cells and the level of protein expression was assessed by quantifying GFP by FACS (Figure 3B). After 48 h, the cells were infected with p24 normalized delta *env* VSV-G pseudotyped NL4–3 and 210WW viruses produced by 293T cells. After an additional 24 h, DNA was extracted, amplified using the sensitive mutation assay for detecting G-to-A mutation in the viral population [17], and sequenced. In SupT1 cells transfected with A3C, G-to-A mutation was predominately in the GA context with 210WW showing significantly higher levels of mutation in comparison to NL4–3 (Figure 3C). No mutation was detected following infection with 210WW or NL4–3 in SupT1 cells transfected with the empty vector (Figure 3C). Therefore, A3C is sufficient to induce mutations in the patient-derived infectious molecular clone of HIV-1.

### The Replication Kinetics of 210WW Are Not Reduced Compared to NL4–3 after Spreading Infection in Cells Expressing A3C

**Figure 4:**
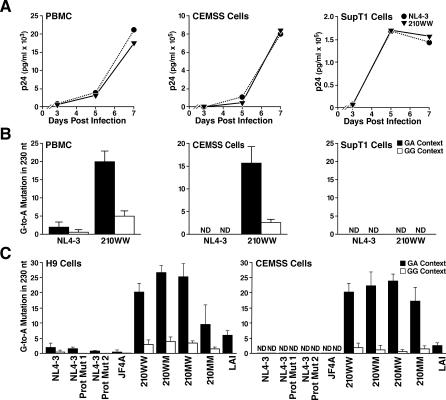
G-to-A Mutation of Wild-Type Virus in PBMC and Different Cell Lines (A) Growth kinetics of 210WW and NL4–3 in PBMC, CEMSS, and SupT1 cells. Cells were infected with p24-normalized viral particles produced from transfected 293T cells. Virus production was monitored by measuring HIV-1 p24 concentration in the culture supernatant. The data presented are one from four independent experiments, in which comparable results were obtained. (B) G-to-A mutation in HIV-1 strains 210WW and NL4–3. DNA was isolated from cell cultures 5 d after infection; the viral protease gene was amplified using the sensitive mutation assay for detecting G-to-A mutated templates. PCR products were then sequenced as a population. The total number of G-to-A mutations in the protease region (positions 2255–2485) was counted separately for the GA and GG contexts. ND, non-detected mutations. (C) Analysis of the GA and GG contexts of the protease gene in the different group of viruses after 7 d of H9 and CEMSS infections. The results presented are the mean and the standard deviations (error bars) of at least three independent infection experiments.

Because 210WW and NL4–3 have different mutation frequencies in the presence of A3C in a setting of single-round infection, we wanted to assess the influence of A3C-induced mutation on the viral replication kinetics in a spreading infection. 293T-derived viruses were normalized by p24 and used to inoculate PBMC, CEMSS, and SupT1 cells, three cell types that have different APOBEC expression profiles. Consistent with previous reports, we found that PBMC expressed A3C, A3G, and A3F [9,16], and that SupT1 was negative for all APOBEC RNA analyzed [27] (Figure 1). In contrast to a previous study [7], we found that CEMSS expressed A3C only (Figure 1). Viral replication kinetics were monitored by measuring HIV-1 p24 concentration in the culture supernatant. We found that 210WW and NL4–3 had comparable growth kinetics in all three cell types (Figure 4A).

The presence and sequence context of G-to-A mutations in NL4–3 and 210WW was next examined in the three cell types at day 5 post infection using the same sensitive mutation assay as above. Frequent G-to-A mutations were found in 210WW sequences extracted from PBMC and CEMSS cells (A3C-positive, Figure 4B). Consistent with A3C activity, they were detected both at GA and GG contexts with GA preferred to GG [7,12]. In contrast, no G-to-A mutation was detected in SupT1 cells (Figure 4B); although the viruses had achieved comparable levels of replication as in the other cells (Figure 4A). NL4–3 sequences showed little to no mutation in any of the three cell types (Figure 4B). Therefore, A3C does not seem to reduce the replication kinetics during spreading infection of 210WW in comparison to NL4–3 despite their difference in A3C susceptibility.

### The Frequency of Lethal Mutations Induced by A3C Is Low in Cells Expressing A3C

To understand why A3C-induced mutations did not reduce the replication kinetics of 210WW we assessed the number of stop codons present in a region of RT using a quantitative assay based on blue and white β-galactosidase complementation (see Materials and Methods). In this assay, the wild-type RT sequence gives rise to a blue colony whereas a mutated sequence containing a stop codon will give rise to a white colony. At day 5 post infection by 210WW, cellular DNA from PBMC, CEMSS, and SupT1 were amplified using primers that cannot distinguish between mutated and non-mutated sequences. The PCR fragments, containing six tryptophan codons susceptible to become stop codons if G-to-A mutations occur, were inserted in frame with the β-galactosidase gene. After validating the assay (see Materials and Methods), we counted the number of white and blue colonies obtained from the different infections. In SupT1 cells infected with 210WW, four out of 1,219 clones were white (0.3%). This rate is equivalent to the background of the assay. Upon sequencing, the few white clones that were found contained insertion-deletion mutations leading to frame-shifts, rather than G-to-A mutations leading to stop codons, consistent with the absence of A3C in SupT1 cells. In contrast, 78 out of 937 clones from PBMC were white (8.3%), and 61 out of 1,290 clones from CEMSS were white (4.7%). The low frequencies of lethal mutations (4%– 8%) induced in A3C-expressing cells could explain the comparable kinetics observed between 210WW, which is susceptible to A3C, and NL4–3, which is relatively resistant.

### The Overall Rate of G-to-A Mutations in the Viral Population Is Low in Cells Expressing A3C

The low frequency of lethal mutation suggests that the overall rate of G-to-A mutation within the viral population should also be low in cells expressing A3C. In order to quantify the overall mutation rate, a clonal analysis of a 230-nt region of *pro* in proviral DNA of 210WW and NL4–3 infection from PBMC was performed. Neutral primers that do not distinguish between mutated and non-mutated sequences were used to amplify 210WW and NL4–3 DNA from PBMC that had been infected 5 d earlier. PBMC infection with NL4–3 *Δvif* virus served as a positive control for G-to-A mutation. Of 45 210WW clones, 33 were wild type and 12 were mutated (27%), with only one to two G-to-A mutations each in the region analyzed. Of 47 NL4–3 clones, 43 were wild type and four were mutated (8%). Of 41 clones of *Δvif* virus, four were wild type and 37 were mutated (90%). We then determined the frequency of mutations leading to lethal premature stop codons: four of 45 (9%) contained premature stop codons consistent with the result of the blue and white β-galactosidase complementation assay and the non-attenuated replication kinetics of 210WW (Figure 4A). Two of 47 (4%) NL4–3 clones had lethal stop codons, and 28 of 41 (68%) *Δvif* clones had lethal stop codons in the *pro* gene.

### G-to-A Mutation May Generate Drug-Resistance Mutations

To investigate whether G-to-A mutation could enhance the capacity of patient-derived viruses to adapt to antiviral drugs, we screened mutated clones for drug-resistance mutations. We looked for *pro* D30N, a mutation in the protease gene that occurs during nelfinavir treatment [28] and is caused by G-to-A mutation in the GA context. Such a modification was found in two of 45 (4%) 210WW clones in the absence of drug selection but in none of the NL4–3 or *Δvif* clones. These results suggest that limited G-to-A mutation could be deleterious for some viral progeny, but surviving progeny may have greater genetic diversity that could allow adaptation to antiviral drugs.

### A3C-Induced G-to-A Mutations Are Not Restricted to 210WW Isolates

To determine if A3C-induced G-to-A mutation occurs in viruses other than 210WW, and to correlate the susceptibility for G-to-A mutation to Gag and/or Pro, we examined G-to-A mutation in a number of viruses. The first group is the 210 virus family (210WW, 210MW, 210WM, and 210MM) that consists of reconstructed HIV-1 molecular clones derived from combinations of pre- and post-therapy *gag* and *pro* genes from a patient who had developed resistance to ritonavir within 4 wk of treatment and that were cloned into a NL4–3 genetic background [26]. These molecular clones demonstrate substantially different phenotypes with respect to drug susceptibility, replication capacity, and Gag cleavage characteristics [26,29,30]. The difference in replication capacity between 210WW and 210WM is 5-fold. The chimeric viruses (MW and WM) have intermediate phenotypes representing a 2.5-fold difference in replication capacity with respect to 210WW [26]. There are 11 amino acid differences between the wild-type Gag in 210WW and the mutant Gag in 210MM as well as two amino differences between wild type and mutant protease (Figure S2). The second group consists of RT- and protease inhibitor–resistant viruses constructed by site-directed mutagenesis of NL4–3 (Protease Mutant 1, 2, and JF4A). NL4–3 and LAI were also used. We repeated the experiments in Figure 4B using an expanded number of viruses from 293T transfections and substituting H9 cells for PBMC (also A3C-, A3G-, and A3F-positive, Figure 1). Population sequencing followed by the dinucleotide context analysis of G-to-A mutations was performed on cellular DNA at day 7 post infection using the G-to-A sensitive detection assay. Preferential (approximately 15-fold) G-to-A mutation in the GA as opposed to the GG context was seen in 210WW, 210WM, 210MW, and 210MM (Figure 4C). Susceptibility to G-to-A mutation was not restricted to the 210 viruses: LAI showed an intermediate to low level of mutation in H9 and CEMSS. G-to-A mutations in LAI also occurred predominantly in the GA context. The 210 viruses showed significantly higher levels of mutation in comparison to the other NL4–3-based and LAI viruses tested in H9 and CEMSS cells (Figure 4C). No mutations were detected in SupT1 cell infections with any of the viruses tested.

### Target Cell Effect of A3C

To determine whether A3C from the producer cell, target cell, or both induces G-to-A mutations, we measured mutations in A3C-positive and A3C-negative target cells from viruses derived from A3C-positive 293T cells directly, or after propagation for 7 d in A3C-negative cells (SupT1). Viral infection titers were normalized by supernatant Gag p24. Infections were carried out and G-to-A mutations were determined as described in Figure 4B. We found no G-to-A mutations in non-expressing A3C target cells infected with 210WW regardless of A3C expression in the producer cells (Table 1). However, G-to-A mutations were induced in target cells expressing A3C. The result suggests that only A3C in the target cell is active.

**Table 1:**
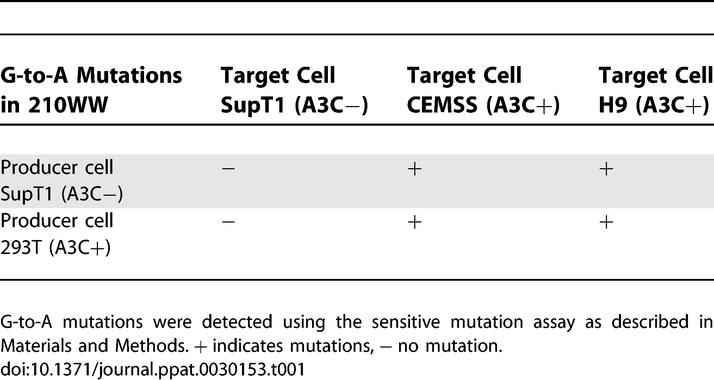
Detection of G-to-A Mutations in 210WW Virus Produced in either A3C-Positive or AC3-Negative Cells following Infection of AC3-Positive and AC3-Negative Cells

## Discussion

In this study, we asked whether A3C has a role in HIV-1 G-to-A mutations. We found that transient A3C expression was sufficient to cause mutation of a patient-derived infectious HIV-1 molecular clone in SupT1 cells. Reciprocally, specific abrogation of A3C expression prevented G-to-A mutation in Magi and 293T cells, demonstrating that A3C is required for mutation in these cell lines. Furthermore, G-to-A mutation is detected in CEMSS cells, which we found express A3C but not A3B, A3F, or A3G. A role for A3C in PBMC is suggested by the activity of the enzyme that can act on both GA and GG contexts: Both mutational patterns are widely observed in vivo with G-to-A mutation in the GA context more predominant than in the GG context [5,6,17]. The preferential activity of A3C on GA context reported by previous studies [7,12,16] is consistent with our findings. Indeed, in CEMSS cells that endogenously express only A3C, G-to-A mutation was predominately detected in the GA context (Figure 4B). These data are consistent with the notion that A3C is necessary and sufficient to induce G-to-A mutation in HIV-1.

G-to-A mutations occurring at low rates in a high background of wild-type viruses were detected in this study using a sensitive G-to-A detection assay adapted from Janini et al, 2000 [17]. Although this approach is useful to sensitively detect G-to-A mutations in the viral population, and to assess the relative frequency of mutation in the GA versus GG context, it is not quantitative. To confirm and quantify the rate of mutation detected in A3C-expressing cells we used the blue and white complementation assay and clonal analysis of sequences.

The blue and white complementation assay and clonal analysis of gene segments amplified using non-selective primers indicated that the majority of viral sequences was not altered after exposure to A3C: 27% of the 210WW clones harbor only one to two G-to-A mutations. We also found that mutated sequences containing premature stop codons induced by G-to-A mutation reflect less than 9% of the 210WW clones. A low rate of G-to-A mutation likely explains the negligible antiviral effect of A3C on the 210 and LAI viruses. Other studies reported that A3C did not exhibit strong antiviral activity against HIV-1 NL4–3 replication, which is consistent with our own observations [7,16]. The minimal antiviral effect of A3C reflects the low frequencies of mutation documented in blue and white complementation assay and clonal analysis of virus populations. Limited G-to-A mutation associated with A3C may be deleterious for some viral progeny, but surviving progeny would have greater genetic diversity. Protease D30N mutations, which confer clinically important resistance to the protease inhibitor nelfinavir [28], appeared in 5% of 210WW clones from PBMC infections. While other proteins expressed in PBMCs may have contributed to the D30N mutations observed, A3C is a likely inducer for the following reasons: A3C is expressed in PBMCs; D30N mutations were observed exclusively in infections with 210WW, which is susceptible to A3C, but not in infections with NL4–3 or NL4–3 *Δvif,* which are susceptible to other deaminases; and all three viruses have the same reverse transcriptase, suggesting that reverse transcriptase errors could not account for the differential rates of mutation observed in the different infections.

The action of A3C was observed primarily in infectious molecular clones of HIV-1, derived from a patient who rapidly developed drug resistance and virological drug failure during therapy with a protease inhibitor [26]. G-to-A mutation associated with A3C expression was also observed in the molecular clone LAI, which may suggest that A3C action is not restricted to the 210 viruses. Susceptibility to A3C-mediated mutation was higher in the 210 family of viruses compared to the NL4–3 family of viruses. The mechanism for this difference in susceptibility to A3C is not known, although the 210 family of viruses differs from NL4–3 in Gag and Pro (the DNA alignment as well as the amino acid alignment of 210WW, LAI, and NL4–3 is provided in Figures S1 and S2). The patient-derived *gag* segment in 210 is 6% divergent from NL4–3 overall. Differences in Gag may alter the stability of the viral core, or the pre-integration complex, which would affect how long nascent single-stranded cDNA is available to bind with A3C in the target cell. More work is needed to identify the viral determinants of A3C susceptibility, and to seek clinical correlations of A3C susceptibility.

The action of A3C differs from that of A3G, A3F, and A3B in several ways. A3G appears to act both in the virion and the target cell: target cell action appears to involve impaired reverse transcription rather than cytidine deamination [31]. A3G, A3F, and A3B cause G-to-A hypermutation when incorporated in the budding virion, producing lethal hypermutation early in the synthesis of viral cDNA that frequently occurs in the virion. In contrast, A3C seems to act only in the target cell based on the following observations: G-to-A mutation was not observed in SupT1 cells (using the sensitive and the blue and white assays) that were infected with viruses produced in 293T cells, which express A3C (Figure 1), while viruses propagated in SupT1 acquire mutation only when they infect A3C-expressing cells (Table 1). Target cell action was further demonstrated by transient expression or transient knockdown of A3C in target cells, which determined the appearance of G-to-A mutation in single-cycle infections. The exposure of the viral cDNA to A3C in the target cell could be limited, which may decrease the frequency of viral mutation to sublethal levels, allowing surviving progeny to have greater genetic diversity to adapt and escape from immune responses or antiviral drugs.

HIV diversity is the major obstacle for effective treatment of HIV patients. It has been suggested that natural genetic variations in A3G/A3F and/or *vif* may result in an incomplete neutralization of those cytidine deaminases allowing the remaining enzymes to exert a low level of mutation on the HIV-1 genome and potentially inducing viral diversity [32,33,34]. Although A3G mutants have been recovered from different individuals, there is no functional evidence for a defect in the antiviral activity of those mutants and in their ability to induce massive hypermutation resulting in defective viruses [33,34]. Similarly, *vif* variants that partially fail to neutralize A3G have been identified; however, the recovered proviral sequences were highly hypermutated and replication-defective [32]. We find evidence of a low level of cytosine deaminase activity in *vif*-expressing viruses, although some Vif-A3C interactions have been observed [7,9,16]. A3C activity may be relatively resistant to Vif [7,19,35,36] because A3C has a tyrosine instead of the aspartic acid at position 128, which is critical for the interaction with and sensitivity to Vif. In conclusion, we demonstrate here that A3C is necessary and sufficient for G-to-A mutations in some HIV-1 strains. A3C-induced mutations occur at levels that allow replication to persist and may therefore play a role in driving viral diversity.

## Materials and Methods

### Cells and viruses.

The cell lines H9, CEMSS, SupT1, Magi, and 293T were directly obtained from the National Institutes of Health AIDS Research and Reference Reagent Program. Cell line identity was confirmed using DNA fingerprinting by PCR amplification with allele-specific primers (Research Genetics). PBMCs from leukocyte-enriched fractions of whole blood from HIV-1-seronegative donors were isolated by density-gradient centrifugation (Histopaque-1077, Sigma). Cells were stimulated with PHA-P (5 μg/ml, Sigma) for 24 h, washed, and maintained at 2 × 10^6^/ml in RPMI-1640 (Irvine Scientific) supplemented with L-glutamine (2 mM), 20% FBS (Gemini Bioproducts), and IL-2 (10 U/ml purified human, Roche Diagnostics) before and after infection.

pNL4–3, obtained from the NIH AIDS Research and Reference Reagent Program (Catalog Number: 114), is a laboratory wild-type HIV-1 molecular clone. pM46I/L63P/V82T/I84V (Catalog Number: 4595), and pL10R/M46I/L63P/V82T/I84V (Catalog Number: 4596) are NL4–3-based infectious molecular clones with protease mutations introduced in vitro. pJF4A (Catalog Number: 1412) is a full-length infectious molecular clone that is identical to pNL4–3 except for two nucleotide substitutions introduced by site-directed mutagenesis at nt 2754 (A-G) and 2755 (C-A) that render it resistant to ddC. pLAI.2 (Catalog Number: 2532) is a laboratory wild-type molecular clone.

The 210 viruses are isogenic infectious molecular clones having the NL4–3 background and combinations of pre-therapy and post-therapy *gag* and *pro* gene segments from a patient who had developed resistance mutations to ritonavir 4 wk after treatment [26]. The 210 constructs differ from NL4–3 in a portion of the 5′ untranslated region upstream of *gag, gag,* and *pro* (nt 702-2563 relative to NL4–3; see Figure S1).

210WW has pre-therapy *gag* and *pro,* while 210MM has drug-adapted *gag* and *pro,* which contains *pro* mutations I54V and V82A. 210WM has the pre-therapy *gag* with drug-adapted *pro,* and 210MW has drug-adapted *gag* with pre-therapy *pro.*

NL4–3 *Δenv* and 210WW *Δenv* viruses were generated by digesting each plasmid with Nhe1 restriction enzyme (Invitrogen). The DNA polymerase I Klenow fragment (New England Biolabs) was used to fill in the ends and the plasmids were religated using the T4 DNA ligase (Promega). Pseudotyped VSV-G ΔEnv NL4–3 and 210WW viruses were produced by cotransfecting 293T with the pMD.G plasmid expressing the VSV-G envelope protein [37] and the plasmid expressing ΔEnv HIV-1 viruses, respectively.

NL43 *Δvif* is a NL4–3 variant with three stop codons introduced into *vif* by directed mutagenesis (C. de Noronha, unpublished data).

### HIV-1 replication and infectivity.

Virus stocks were prepared by Lipofectamine-mediated transfection of 293T cells. The clarified viral supernatants were quantified and normalized by HIV-1 Gag p24 ELISA (Perkin Elmer). DNase treatment of viral supernatant was carried out at 37 °C for 30 min with the equivalent of 10 units DNase (Sigma)/1 ml of the clarified crude virus. Spreading infections were monitored over time by accumulation of p24 Gag in the culture supernatants. Single-cycle infectivity was performed by challenging the cell lines (5 × 10^5^ cells) with VSV-G *Δenv* pseudotyped viruses for 24 h.

### APOBEC plasmid construction.

PolyA+ RNA was isolated from cells with the QuickPrep Micro mRNA Purification Kit (Pharmacia). The open reading frames of A3B, A3C, A3F, and A3G were amplified by RT-PCR with the following primers: 3B forward 5′-ATGAATCCACAGATCAGAAATCC-3′ and reverse 5′-TCAGTTTCCCTGATTCTGG-3′; 3C: forward 5′-ATGAATCCACAGATCAGAAACC-3′ and reverse 5′-TCACTGGAGACTCTCCCGTA-3′; 3F: forward 5′-ATGAAGCCTCACTTCAGAAAC-3′ and reverse 5′-TCACTCGAGAATCTCCTGC-3′; 3G: forward 5′-ATGAAGCCTCACTTCAGAAACACAG-3′ and reverse 5′-TCAGTTTTCCTGATTCTGGAGAATGG-3′. The amplicons were cloned into pGEM-T easy vectors (Promega) and confirmed by sequencing. The A3C gene with HA tag sequence at its 3′ terminus was amplified from mRNA of H9 cells by RT-PCR, and the identity of the product was confirmed by sequencing. It was then cloned into pTT-IRES-GFP (a gift from David Fenard, Gladstone Institute of Virology and Immunology) for protein expression.

### Transfection.

The 293T or Magi cells were transfected with Lipofectamine 2000 transfection reagent (Invitrogen), according to the manufacturer's recommendations. The SupT1 cells were transfected by Amaxa electroporation technology with the program T29, according to the manufacturer's recommendations.

### Nucleic acid isolation, RT, PCR, and DNA sequencing.

DNA was extracted from infected cells with the DNA Easy kit (Invitrogen) and quantified by spectrometry. PolyA + RNA was isolated with QuickPrep Micro mRNA Purification kit (Pharmacia). Reverse transcription reaction was performed with random hexamers using SuperScript III reverse transcriptase (Invitrogen). An RT minus control was always performed in parallel to check for DNA contamination. PCR was performed with Easy-A High Fidelity PCR Cloning Enzyme (Stratagene). APOBEC reading frames (A3B, A3C, A3F, and A3G) were amplified with primers specific for a single gene and listed above in the APOBEC plasmid construction section. The specificity of the primers was verified by sequencing the PCR products. The PCR products were sequenced by dye terminator cycle sequencing with Big Dye v3.1 (Applied Biosystems) or ET Terminators (Amersham Biosciences) on an ABI 3100 microcapillary sequencer. Raw sequence data was analyzed and aligned using Sequencher (Gene Codes).

### Sensitive mutation assay for the detection of G-to-A mutation.

This assay is designed to detect G-to-A viral mutated sequences present in a high wild-type background. A nested PCR procedure specific for a 230-nt region in the protease gene amplifies both G (wt) and A (mut) species in the GA and GG context using the HIV-1 *pro* primers Hypa 10 and Hypa 11 in the first PCR and DP 16 and DP 17 in the second round PCR as described [17].

The first round PCR uses primers specific for the mutated sequences (Hypa 10 and Hypa 11). The goal is to enrich the mutated sequences that are usually present in low concentration. The second round PCR uses primers that can amplify both mutated (if present from the first round PCR) and the input wild-type DNA (carried over from the first PCR) [17]. Under these amplification conditions, no PCR-induced mutations were detected using control plasmids as templates. Protease sequences were determined as population sequences. A mixture of G and A peaks was typically seen at the mutation site. A change from G-to-A was considered a true mutation only if A represented at least 20% of the mixture.

### Clonal analysis.

The clonal analysis was performed using the neutral primers DP16–2 and DP17–2. The sequence of DP16–2 is 5′-TATCCTTTAGCTTCCCTCA-3′. The sequence of DP17–2 is 5′-TAATGGGAAAATTTAAAGTGCAG-3′. PCR products were cloned in pCR 4-TOPO (Invitrogen). The plasmids were then sequenced and analyzed for their G-to-A content.

### Blue and white beta galactosidase complementation assay.

The genetic assay was carried out with a 111-nucleotide viral DNA containing a HIV-1 *pol* fragment that includes six tryptophan codons susceptible to become stop codons if G-to-A mutations occur. Viral DNA was amplified using the primers Nsi-Trp6 Fw: 5′-CCA ATGCAT**ACTCCTAAAT TTAAATTACC CATACAAAA**-3′ and Trp6 Rev Spe: 5′-GACTAGT**TAAGGGAGGG GTATTGA-3′.** The amplified DNA was phenol extracted, precipitated with ethanol, subjected to treatment with Nsi1 and SpeI, and then purified from a 1% agarose gel. This fragment encoding six Trp codons was cloned in frame into the *lacZ* fragment of the pGEM-T vector. For this purpose pGEM-T was digested first with Nsi1 and then with SpeI to obtain the linearized vector, which was purified from a 1% agarose gel and treated with alkaline phosphatase before ligation to the PCR products. Escherichia coli XL-1 Blue was transformed and plated onto minimal agar plates containing 8% X-Gal (5-bromo-4-chloro-3-indolyl-d-galactopyranoside) and 20% IPTG (isopropyl-d-thiogalactopyranoside). The plates were incubated at 37 °C for approximately 15 h. We validated the assay by first mixing 0%–5%–25%–50%–75%–100% of G-to-A mutated clones with wild-type clones. We then amplified each mix, cloned the PCR product, and counted the number of blue and white colonies. We confirmed that the number of blue and whites clones obtained after every specific mixture with the level of mutated sequences included in the mix. We also determined that the background of this assay was around 0.3%, which corresponded to the percentage of white colonies detected using 100% wild-type plasmids. The sequences of the white clones obtained in this specific mix revealed insertions or deletions interrupting the reading frame, and not stop codons. We also sequenced 20 white and 20 blue clones obtained from the other mix to verify their mutation content and the presence of stop codons. 20/20 blue clones were wild-type sequences and 20/20 white clones were mutated sequences. Blue and white clones were counted using the EagleSight (Stratagene) instrument and confirmed by manual counting.

### Western blot analysis.

A3C protein expression in 293T transfected cells was detected by western blot with an HA-probe (Y-11) and HRP polyclonal antibody specific for the HA tag (Santa Cruz Biotechnology).

### RNAi assay.

siRNAs targeting A3C from positions 167–185 (siRNA 1) and 107–125 (siRNA 2) bases relative to the start codon were transcribed in vitro (RiboMax kit, Promega) from an oligo DNA template containing the T7 promoter synthesized by Invitrogen. The sequence of the scrambled RNA for both siRNAs was generated with the Promega siRNA target designer.

For the siRNA transfection, 293T and Magi cells were seeded into six-well plates at a density of 10^4^ cells/ml. After about 2 h, purified A3C siRNAs along with RNA oligo conjugated to FITC (Block It kit, Invitrogen) were transfected into the cells with Lipofectamine 2000, according to the manufacturer's protocols. A scrambled siRNA was transfected separately as a control.

The efficiency of transfection was monitored by fluorescent microscopy at 24, 48, and 72 h after siRNA transfection. For flow cytometry analysis, the cells were analyzed with a Becton Dickinson FACS cytometer. Samples were counted and analyzed with FlowJo software (Tree Star). Non-transfected cells were used as a control. FITC-positive cells were sorted (DIVA instrument, Becton Dickinson) for further analysis and infection.

## Supporting Information

Figure S1 Nucleic Acid Alignment of 210WW, 210MM, NL4–3, and LAI Viruses The alignment includes the segment of the 5′ untranslated region upstream of *gag, gag,* and *pro* (nt 702-2563 relative to NL4–3). The dots indicate the nucleotides that match.
(704 KB AI).

Figure S2 Amino Acid Alignment of 210WW, 210MM, NL4–3, and LAI Viruses (A) Gag amino acid alignment of 210WW, 210MM, NL4–3, and LAI viruses. Amino acid position relative to polyprotein start in HXB2: 1–513. Residue numbers for each line of sequence are provided. The dots indicate the residues that match.
(B) Protease alignment of 210WW, 210MM, NL4–3, and LAI viruses. Amino acid position relative to polyprotein start in HXB2: 557–655.
(500 KB AI).

Figure S3 Partial Reduction of A3C Expression and G-to-A Mutation by siRNA 2 (A) 293T cells were transfected with siRNA 2 (50 nM) against A3C or with scrambled RNA. A FITC-conjugated oligo was cotransfected and FITC-positive cells were sorted 48 h following transfection. PolyA+ RNA was isolated, and RT-PCR was performed with primers specific for A3B (lanes 1 and 2), A3C (lanes 3 and 4), A3F (lanes 5 and 6), and A3G (lanes 7 and 8). β-Actin (lanes 9 and 10) was used as an internal control. A sample lacking template DNA (lane 11) was used as a negative control. Lane 12 is a molecular weight standard.
(B) The transfected cells were infected with the VSV-G-pseudotyped 210WW and collected 24 h later. Viral DNA was amplified using the sensitive mutation assay and population sequencing was performed to analyze G-to-A mutation. The G-to-A mutation typically appeared as a mixture of G and A peaks at a given position, with G, the wild-type sequence, as the predominant peak. A change from G-to-A was considered a true mutation only if A represented at least 20% of the peak.
(C) Base composition of 210WW plasmid, 210WW DNA infection of 293T + scrambled RNA, and 210WW DNA infection of 293T + siRNA 2 in the region amplified by the sensitive mutation assay.
(786 KB AI).

### Accession Numbers

The GenBank (http://www.ncbi.nlm.nih.gov/Genbank/index.html) accession numbers for the genes and proteins mentioned in the text are: A3B (NM_004900), A3C (NM_014508), A3F (NM_001006666), A3G (NM_021822), LAI *gag-pro* (K02013), NL4–3 *gag-pro* (AF324493), 210MM *gag-pro* (EU100418), and 210WW *gag-pro* (EU100417).

## Abbreviations

A3B: APOBEC3B
A3C: APOBEC3C
A3F: APOBEC3F
A3G: APOBEC3G
HA: hemaggluttinin
RT: reverse transcriptase
Vif: virion infectivity factor
