# Supplementary material for: Target Cell APOBEC3C Can Induce Limited G-to-A Mutation in HIV-1
Source: PLoS Pathog. 2007 Oct 26;3(10):e153. doi: 10.1371/journal.ppat.0030153 (PMC2042017; doi:10.1371/journal.ppat.0030153)
Supplement: Figure S1 — The alignment includes the segment of the 5′ untranslated region upstream of gag, gag, and pro (nt 702-2563 relative to NL4–3). The dots indicate the nucleotides that match. (704 KB AI). [file ppat.0030153.sg001.pdf]

07.210 C K.Bourara

```
210WW      1 CTTGCTGAAGCGCGCACGGCAAGAGGCGAGGGGCGGCGACTGGTGAGTACGCCAAAACTT 60
210MM      1 ............................................................ 60
NL4-3      1 .........................................................A.. 60
LAI        1 .................................A.......................A.. 60

210WW     61 TTGACTAGCGGAGGCTAGAAGGAGAGAGATGGGTGCGAGAGCGTCGGTATTAAGCGGGGG 120
210MM     61 ...................................T........................ 120
NL4-3     61 ............................................................ 120
LAI       61 .............................................A.............. 120

210WW    121 AAAATTAGATAAATGGGAGAAAATTCGGTTACGGCCAGGGGGAAAGAAAAAATATAAATT 180
210MM    121 ............................................................ 180
NL4-3    121 .G................A............A.................C........C. 180
LAI      121 .G........CG......A............A............................ 180

210WW    181 AAAACATCTAGTATGGGCAAGCAGGGAGTTAGAACGATTCGCAGTCAATCCTGGCCTTTT 240
210MM    181 ................................................C........... 240
NL4-3    181 .......A....................C................T.............. 240
LAI      181 .......A....................C................T...........G.. 240

210WW    241 AGAGACATCAGAAGGCTGTAGACAAATATTGGAACAGCTACAACCATCCCTTAAGACAGG 300
210MM    241 ...........G................A.................G............. 300
NL4-3    241 ............................C...G...................C....... 300
LAI      241 ...A........................C...G...................C....... 300

210WW    301 ATCAGAAGAACTTAGATCATTATTCAATGCAGTAGCAACCCTCTATTGTGTGCATCAAGG 360
210MM    301 ........................T................................... 360
NL4-3    301 .......................AT...A..A.....GT...................A. 360
LAI      301 .......................AT...A.............................A. 360

210WW    361 GATAGATGTAAAAGACACCAAGGAAGCTTTAGACAAGATAGAGGAAGAACAAAACAAAAG 420
210MM    361 ...........G................................................ 420
NL4-3    361 ...........................C.....T..............G........... 420
LAI      361 ......GA........................................G........... 420

210WW    421 TAAGAAAAAGGCACAGCAAGCAGCAGCTGACACAGGAAGCTGCAGCAGCCAAGTCAGCCA 480
210MM    421 ......................................A..................... 480
NL4-3    421 ......................................A---..A......G........ 477
LAI      421 .........A...........................CA---.........G........ 477

210WW    481 AAATTACCCTATAGTGCAGAATCTACAGGGGCAAATGGTACATCAGGCCCTATCACCTAG 540
210MM    481 .....................C...................................... 540
NL4-3    478 .....................C..C........................A.......... 537
LAI      478 .....................CA.C........................A.......... 537

210WW    541 AACATTAAATGCATGGGTAAAAGTAATAGAAGAGAAGGCTTTCAGCCCAGAAGTAATACC 600
210MM    541 G........................................................... 600
NL4-3    538 ...T.....................G.................................. 597
LAI      538 ...T.....................G............................G..... 597

210WW    601 CATGTTTTCAGCATTATCAGAAGGAGCCACCCCACAAGATTTAAACACCATGCTAAACAC 660
210MM    601 ............................................................ 660
NL4-3    598 .............................................T.............. 657
LAI      598 ............................................................ 657

210WW    661 AGTAGGGGGACACCAGGCAGCCATGCAAATGTTAAAAGAGACCATCAATGAAGAGGCTGC 720
210MM    661 ...............A.............................T........A..... 720
NL4-3    658 ...G........T..A...................................G..A..... 717
LAI      658 ...G........T..A...................................G..A..... 717

210WW    721 AGAATGGGATAGATTGCATCCAGTGCATGCAGGGCCTATTGCACCAGGCCAGATGAGAGA 780
210MM    721 ............................................................ 780
NL4-3    718 ............................................................ 777
LAI      718 .............G.............................................. 777

210WW    781 ACCAAGGGGAAGTGACATAGCAGGGACTACTAGCACCCTTCAGGAACAAATAGGATGGAT 840
210MM    781 ...............T............................................ 840
NL4-3    778 ........................A........T.......................... 837
LAI      778 ........................A........T.......................... 837

210WW    841 GACACATAATCCACCTATCCCAGTAGGAGAAATCTATAAAAGATGGATAATCCTGGGACT 900
210MM    841 ............................................................ 900
NL4-3    838 ..........................................................T. 897
LAI      838 ....A............................T........................T. 897

210WW    901 AAATAAAATAGTAAGAATGTATAGCCCTGTCAGCATTCTGGACATAAGACAAGGGCCAAA 960
210MM    901 .....................C....................T................. 960
NL4-3    898 ............................AC........................A..... 957
LAI      898 ............................AC........................A..... 957

210WW    961 GGAACCATTTAGAGATTATGTAGACAGGTTCTATAAAACTTTAAGAGCCGAGCAAGCTTC 1020
210MM    961 .........................C.................................. 1020
NL4-3    958 ......C........C.........C.A............C................... 1017
LAI      958 A.....C........C.........C..............C................... 1017

210WW   1021 ACAGGAGGTAAAAAATTGGATGACAGAAACCTTGTTGGTCCAAAATGCGAACCCAGATTG 1080
210MM   1021 ............................................................ 1080
NL4-3   1018 ...A........................................................ 1077
LAI     1018 ............................................................ 1077

210WW   1081 TAAGACTATCTTAAAAGCATTGGGACCAGCAGCTACACTAGAAGAAATGATGACAGCATG 1140
210MM   1081 ............................................................ 1140
NL4-3   1078 .........T...................G...G.......................... 1137
LAI     1078 .........T.................................................. 1137

210WW   1141 CCAGGGAGTGGGGGGACCTGGCCATAAAGCAAGAGTTTTGGCTGAAGCAATGAGCCAAGT 1200
210MM   1141 ............................................................ 1200
NL4-3   1138 T.................C......................................... 1197
LAI     1138 T...........A.....C........G................................ 1197

210WW   1201 AACAAATTCAGCTGCCATAATGATGCAGAAAGGTAACTTTAAAGGCCAAAGAAAAATTGT 1260
210MM   1201 ...............................A.C...................GG..... 1260
NL4-3   1198 .......C.....A..........A........C..T....GGAA.........G.C... 1257
LAI     1198 .............A.............A.G...C..T....GGAA.........G..... 1257

210WW   1261 TAAGTGCTTCAATTGTGGCAGAGAAGGGCACATAGCCAAAAATTGCAGGGGCCCTAGGAA 1320
210MM   1261 ......T...........................................C......A.. 1320
NL4-3   1258 ......T.............A.............................C......... 1317
LAI     1258 ......T.............A.................G...........C......... 1317

210WW   1321 AAAGGGCTGTTGGAAATGTGGAAAGGAAGGACACCAAATGAAAGATTGTACTGAGAGACA 1380
210MM   1321 ................................................C.T......... 1380
NL4-3   1318 ............................................................ 1377
LAI     1318 ............................................................ 1377

210WW   1381 GGCTAATTTTTTAGGGAAGCTCTGGCCTTCCAACAAGGGGAGGCCAGGGAATTTTCTCCA 1440
210MM   1381 ..T......................................................T.. 1440
NL4-3   1378 ...................A...........C.......A.................T.. 1437
LAI     1378 ...................A...........T.......A.................T.. 1437

210WW   1441 GAGCAGACCAGAGCCAACAGCCCCACCA-------------------------------- (1468)
210MM   1441 ......G.....................-------------------------------- (1468)
NL4-3   1438 ............................-------------------------------- (1465)
LAI     1438 ............................TTTCTTCAGAGCAGACCAGAGCCAACAGCCCC 1497

210WW (1469) ----GAAGAGAGCTTCAGGTTTGGGGAAGAGACAACAACTCCCACTCAGAAGCAGGAGCC 1524
210MM (1469) ----.......................G................................ 1524
NL4-3 (1466) ----.......................................T................ 1521
LAI     1498 ACCA................C.....T................T................ 1557

210WW   1525 TCTAGACAAGGAACTGTATCCTTTAGCTTCCCTCAGATCACTCTTTGGCAACGACCCCTC 1584
210MM   1525 ............................................................ 1584
NL4-3   1522 GA................................................G......... 1581
LAI     1558 GA..............--------------------........................ 1617

210WW   1585 GTCACAATAAAGATAGGGGGGCAACTAAAGGAAGCTCTATTAGACACAGGAGCAGATGAT 1644
210MM   1585 ............................................................ 1644
NL4-3   1582 ........................T...................T............... 1641
LAI     1618 ............................................T............... 1677

210WW   1645 ACAGTATTAGAAGAAATGAATTTGCCAGGAAGATGGAAACCAAAAATGATAGGGGGAATT 1704
210MM   1645 ............................................................ 1704
NL4-3   1642 ............................................................ 1701
LAI     1678 ...................G........................................ 1737

210WW   1705 GGAGGTTTTATCAAAGTAAGACAGTATGATCAGATACCAATAGAAATCTGCGGACATAAA 1764
210MM   1705 ............................................................ 1764
NL4-3   1702 .....................................TC..................... 1761
LAI     1738 .....................................TC..........T......... 1797

210WW   1765 GCTATAGGTACAGTTTTAGTAGGACCTACACCTGTCAACATAATTGGAAGAAATCTGTTG 1824
210MM   1765 ............................................................ 1824
NL4-3   1762 ..............A............................................. 1821
LAI     1798 ..............A............................................. 1857

210WW   1825 ACTCAGATTGGCTGCACTTTAAATTTT 1851
210MM   1825 ........................... 1851
NL4-3   1822 ........................... 1848
LAI     1858 ...........T............... 1884
```
