# Supplementary material for: Target Cell APOBEC3C Can Induce Limited G-to-A Mutation in HIV-1
Source: PLoS Pathog. 2007 Oct 26;3(10):e153. doi: 10.1371/journal.ppat.0030153 (PMC2042017; doi:10.1371/journal.ppat.0030153)
Supplement: Figure S2 — (A) Gag amino acid alignment of 210WW, 210MM, NL4–3, and LAI viruses. Amino acid position relative to polyprotein start in HXB2: 1–513. Residue numbers for each line of sequence are provided. The dots indicate the residues that match. (B) Protease alignment of 210WW, 210MM, NL4–3, and LAI viruses. Amino acid position relative to polyprotein start in HXB2: 557–655. (500 KB AI). [file ppat.0030153.sg002.pdf]

A Gag Sequences

```
210WW   1 MGARASVLSGGKLDKWEKIRLRPGGKKKYKLKHLVWASRELERFAVNPGLLETSEGCRQI 60
210MM   1 ..V...................................................G..... 60
NL4-3   1 ...........E...............Q.....I.......................... 60
LAI     1 ...........E..R..................I.......................... 60

210WW  61 LEQLQPSLKTGSEELRSLFNAVATLYCVHQGIDVKDTKEALDKIEEEQNKSKKKAQQAAA 120
210MM  61 M.....A...........................R......................... 120
NL4-3  61 .G......Q.........Y.TI.V......R............................. 120
LAI    61 .G......Q.........Y.T.........R.EI.......................... 120

210WW 121 DTGSCSSQVSQNYPIVQNLQGQMVHQALSPRTLNAWVKVIEEKAFSPEVIPMFSALSEGA 180
210MM 121 ...N........................................................ 180
NL4-3 121 ...N-N.....................I...........V.................... 179
LAI   121 ...H-.............I........I...........V.................... 179

210WW 181 TPQDLNTMLNTVGGHQAAMQMLKETINEEAAEWDRLHPVHAGPIAPGQMREPRGSDIAGT 240
210MM 181 ............................................................ 240
NL4-3 180 ............................................................ 239
LAI   180 ...................................V........................ 239

210WW 241 TSTLQEQIGWMTHNPPIPVGEIYKRWIILGLNKIVRMYSPVSILDIRQGPKEPFRDYVDR 300
210MM 241 ............................................................ 300
NL4-3 240 ........................................T................... 299
LAI   240 ............N...........................T................... 299

210WW 301 FYKTLRAEQASQEVKNWMTETLLVQNANPDCKTILKALGPAATLEEMMTACQGVGGPGHK 360
210MM 301 ............................................................ 360
NL4-3 300 ........................................G................... 359
LAI   300 ............................................................ 359

210WW 361 ARVLAEAMSQVTNSAAIMMQKGNFKGQRKIVKCFNCGREGHIAKNCRGPRKKGCWKCGKE 420
210MM 361 .....................S......R..................A............ 420
NL4-3 360 .............P.T..I.....RN...T.......K.........A............ 419
LAI   360 ...............T....R...RN...........K.....R...A............ 419

210WW 421 GHQMKDCTERQANFLGKLWPSNKGRPGNFLQSRPEPTAPP------------EESFRFGE 468
210MM 421 .......I...V............................------------........ 468
NL4-3 420 .................I...H..................------------........ 467
LAI   420 .................I...Y..................FLQSRPEPTAPP.....S.V 479

210WW 469 ETTTPTQKQEPLDKELYPLASLRSLFGNDPSSQ 501
210MM 469 ................................. 501
NL4-3 468 .....S.....I...............S..... 500
LAI   480 .....S.....I.......T............. 512
```

B Protease Sequences

```
210WW   1 PQITLWQRPLVTIKIGGQLKEALLDTGADDTVLEEMNLPGRWKPKMIGGIGGFIKVRQYD 60
210MM   1 .....................................................V...... 60
NL4-3   1 ............................................................ 60
LAI     1 ....................................S....................... 60

210WW  61 QIPIEICGHKAIGTVLVGPTPVNIIGRNLLTQIGCTLNF 99
210MM  61 .....................A................. 99
NL4-3  58 ..L.................................... 99
LAI    58 ..L.................................... 99
```

07.210 B K.Bourara
