# Supplementary figures and images for: Target Cell APOBEC3C Can Induce Limited G-to-A Mutation in HIV-1
Source: PLoS Pathog. 2007 Oct 26;3(10):e153. doi: 10.1371/journal.ppat.0030153 (PMC2042017; doi:10.1371/journal.ppat.0030153)

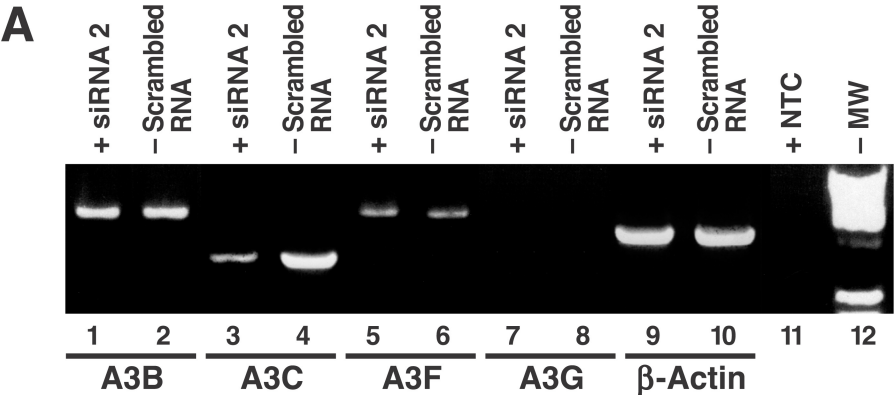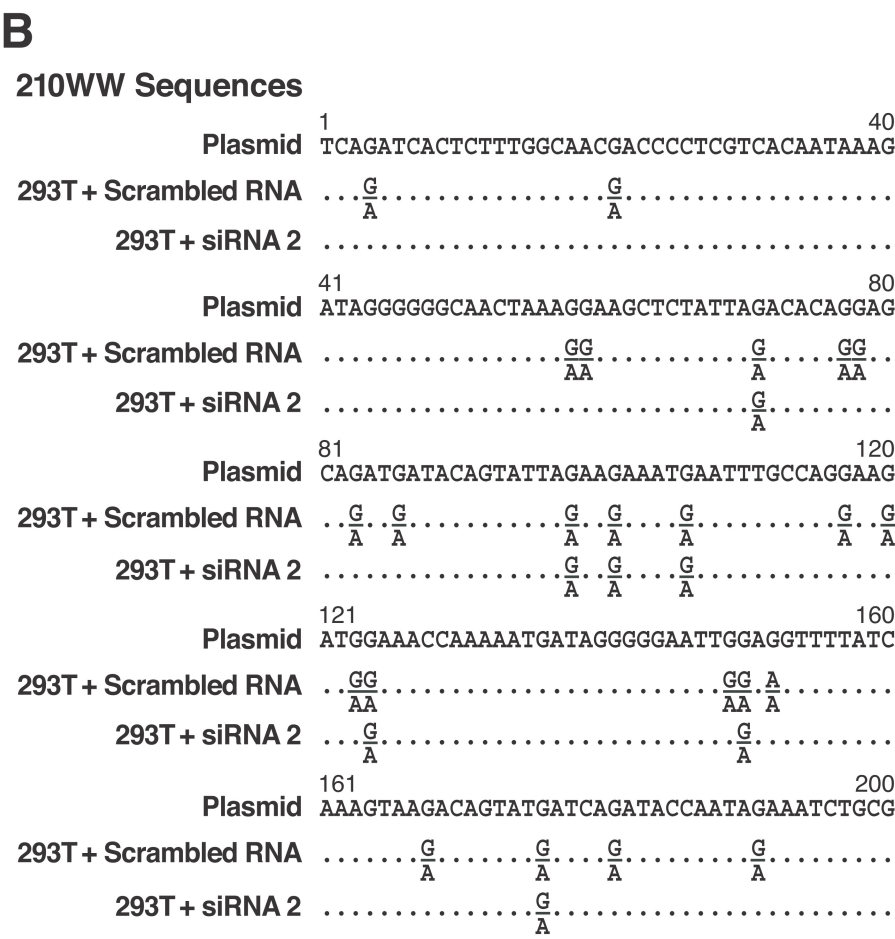

**C**

| Base Composition | Plasmid | 293T + Scr. RNA | 293T + siRNA 2 |
|------------------|---------|-----------------|----------------|
| A                | 77      | A 100           | A 84           |
| C                | 32      | C 32            | C 32           |
| G                | 49      | G 26            | G 42           |
| T                | 42      | T 42            | T 42           |

Supplement: Figure S3 — (A) 293T cells were transfected with siRNA 2 (50 nM) against A3C or with scrambled RNA. A FITC-conjugated oligo was cotransfected and FITC-positive cells were sorted 48 h following transfection. PolyA+ RNA was isolated, and RT-PCR was performed with primers specific for A3B (lanes 1 and 2), A3C (lanes 3 and 4), A3F (lanes 5 and 6), and A3G (lanes 7 and 8). β-Actin (lanes 9 and 10) was used as an internal control. A sample lacking template DNA (lane 11) was used as a negative control. Lane 12 is a molecular weight standard. (B) The transfected cells were infected with the VSV-G-pseudotyped 210WW and collected 24 h later. Viral DNA was amplified using the sensitive mutation assay and population sequencing was performed to analyze G-to-A mutation. The G-to-A mutation typically appeared as a mixture of G and A peaks at a given position, with G, the wild-type sequence, as the predominant peak. A change from G-to-A was considered a true mutation only if A represented at least 20% of the peak. (C) Base composition of 210WW plasmid, 210WW DNA infection of 293T + scrambled RNA, and 210WW DNA infection of 293T + siRNA 2 in the region amplified by the sensitive mutation assay. (786 KB AI). [file ppat.0030153.sg003.pdf]
